# Supplementary material for: Competing magnetic ground states and their coupling to the crystal lattice in CuFe2Ge2
Source: Sci Rep. 2016 Oct 14;6:35325. doi: 10.1038/srep35325 (PMC5064417; doi:10.1038/srep35325)
Supplement: Supplementary Information [file srep35325-s1.pdf]

Supplementary Information: Competing magnetic ground states and their coupling to the crystal lattice in $CuFe_2Ge_2$

Andrew F. May[1,*], Stuart Calder[2], David Parker[1], Brian C. Sales[1], and Michael A. McGuire[1]

[1]Materials Science and Technology Division, Oak Ridge National Laboratory, Oak Ridge, TN 37831, US
[2]Quantum Condensed Matter Division, Oak Ridge National Laboratory, Oak Ridge, TN 37831, USA
[*]mayaf@ornl.gov

ABSTRACT

This Supplementary Information covers sample synthesis and thermal stability of $CuFe_2Ge_2$. Electrical and thermal transport data are also reported, along with additional results from first principles calculations, and x-ray and neutron diffraction data and analysis. Magnetization and x-ray diffraction data for the cobalt doped $CuFeCoGe_2$ sample are included as well.

Synthesis and Phase Stability

The synthesis of $CuFe_2Ge_2$ can be achieved by arc-melting and annealing, and this study demonstrates that the annealing temperature utilized influences the observed properties. Specifically, we find that samples annealed at 600 °C have an x-ray diffraction pattern that is well described by the reported structure and does not contain any significant impurities. This is consistent with the original report by Zavalij.[1] A single impurity peak can sometimes be observed in the laboratory x-ray diffraction data, and can be indexed to Cu_5Ge_2. Samples annealed at higher temperatures do not appear to be phase pure or of the same quality, which is here linked to disorder and/or decomposition.

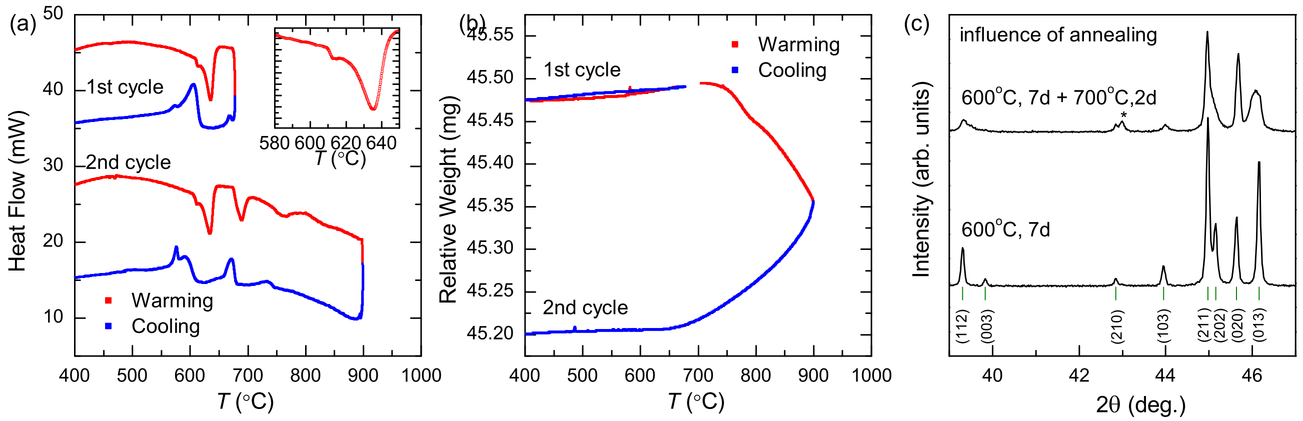

Figure S1. Thermogravimetric analysis and x-ray diffraction showing phase decomposition and/or disordering above $\approx$ 635°C.

Differential thermal analysis (Fig. S1) revealed thermal anomalies at 612, 635, 685, and 750 °C, which were present upon cooling with a clear hysteresis and a little broadening. The thermal anomalies remained upon heating the sample a second time. Heating the $\approx$45 mg sample above $\approx$750 °C produced measurable weight loss, likely due to formation of a volatile oxide upon heating. Measurements were performed under flowing argon.

The events associated with the thermal anomalies shown in Fig. S1 clearly affect the sample quality. The influence of annealing at higher temperatures is shown in Fig. S1c, where powder x-ray diffraction data are shown in a characteristic region (common impurities Cu_5Ge_2, $Fe_{2-x}Ge$, Ge could all be observed in this region) . A sample was formed in the reported way

with a final step of annealing at 600 °C for 7 d, and this sample has well-defined Bragg peaks that are easily indexed to the known structure. A portion of this sample was then annealed for 2 d at 700 °C. After annealing at 700 °C, the diffraction peaks with an L component (H,K,L Miller indexes) are greatly broadened and an impurity peak near 43 degrees 2Θ becomes evident (most likely Cu_5Ge_2). The broadening of certain diffraction peaks would suggest disordering occurs, though the emergence of another peak clearly suggests a chemical change or decomposition. On the contrary, the observation of thermal anomalies upon cooling (Fig. S1a) would suggest a reversible transition occurs, such as disordering or dissolution.

These results for phase stability have implications regarding crystal growth. Crystals with magnetic properties similar to those of the polycrystalline samples annealed at 700 °C can be obtained using a self-flux (excess Cu and Ge). However, these crystals grow at temperatures above the 'decomposition' temperature, and thus it is not clear if they are stoichiometric at the composition $CuFe_2Ge_2$. This is further complicated by the fact that the growth occurs in an environment with excess Cu and Ge. Additional annealing and growth studies are required to fully understand the nature of the obtained crystals. Searching for an inert (molten-metal) flux may be a better route to grow $CuFe_2Ge_2$ crystals below ≈630 °C.

Physical Property Data

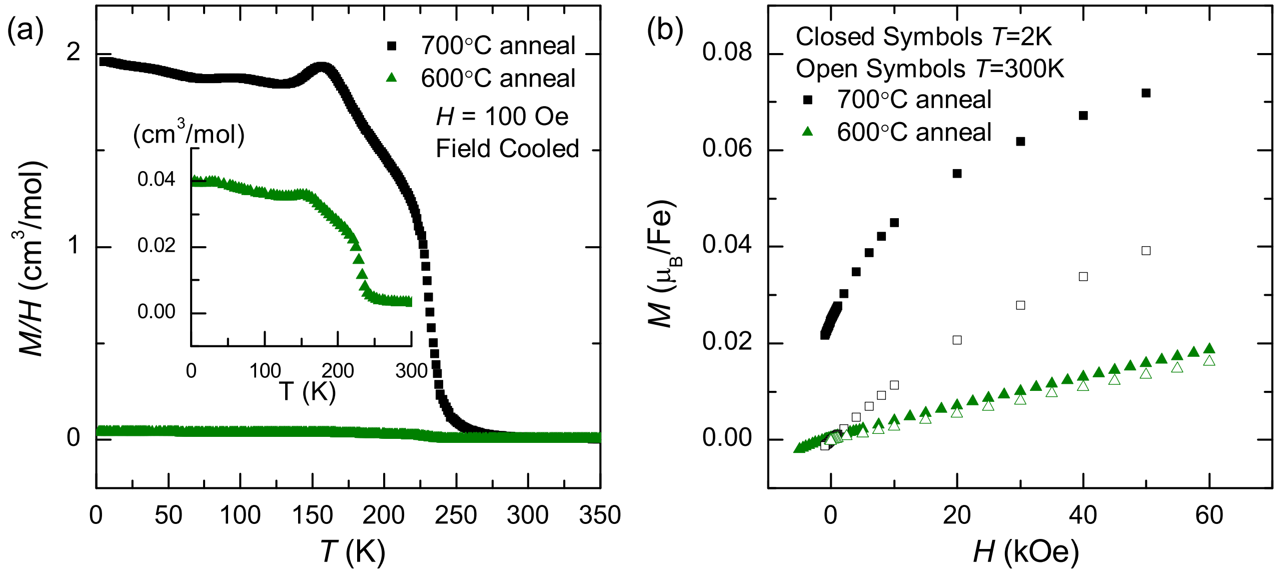

Figure S2. Influence of annealing temperatures on magnetization data; legend labels 600 °C and 700 °C refer to sample annealing conditions as shown in Figure S1. After annealing at 700 °C the induced magnetization increases, as does the remanent magnetization observed at low T. The inset of (a) contains the same data as in the main panel on a scale that allows the temperature-dependence of M to be observed.

Magnetization measurements were performed to further probe the changes that occur upon annealing at 700 °C (See Fig. S2). These results clearly show an increase in the induced magnetization following annealing at 700 °C. Interestingly, the temperature dependence of the induced magnetization M remains qualitatively similar to that observed for the higher-quality samples produced by annealing at 600 °C. As shown in Fig. S2b, the increased magnetization is present as an increase in the ferromagnetic contribution. That is, the remanent moment M_r is significantly larger in the sample that was annealed at 700 °C (at 2 K M_r=0.025μ_B/Fe versus 0.00046μ_B/Fe for annealing at 600 °C). One other way to compare the ferromagnetic contribution is to perform a linear fit to the high field data and compare the M intercept. Fitting between 3 and 5 T yields zero-field (intercept) moments of 0.047μ_B/Fe and 0.0016μ_B/Fe at 2 K for the samples annealed at 700 and 600 °C, respectively. Given these results, it is tempting to conclude that the intrinsic behavior of crystalline $CuFe_2Ge_2$ is an antiferromagnetic ground state with no ferromagnetic contribution (zero remanent moment, zero intercept of the $M-H$ curve from high field). However, the potential for canting of moments in this relatively complex magnetic structure cannot be ignored, nor can the potential of the field itself to modify the magnetic structure. Regardless, though, it is clear that high quality $CuFe_2Ge_2$ should have a nearly linear $M(H)$ curve at low T and display only a very small ferromagnetic contribution.

To produce a dense compact for transport measurements, an arc-melted ingot was ground and hot-pressed at approximately 585°C followed by annealing at 600°C. Hot-pressing occurred in a graphite furnace using a graphite die ($\frac{3}{8}$" diameter) with an

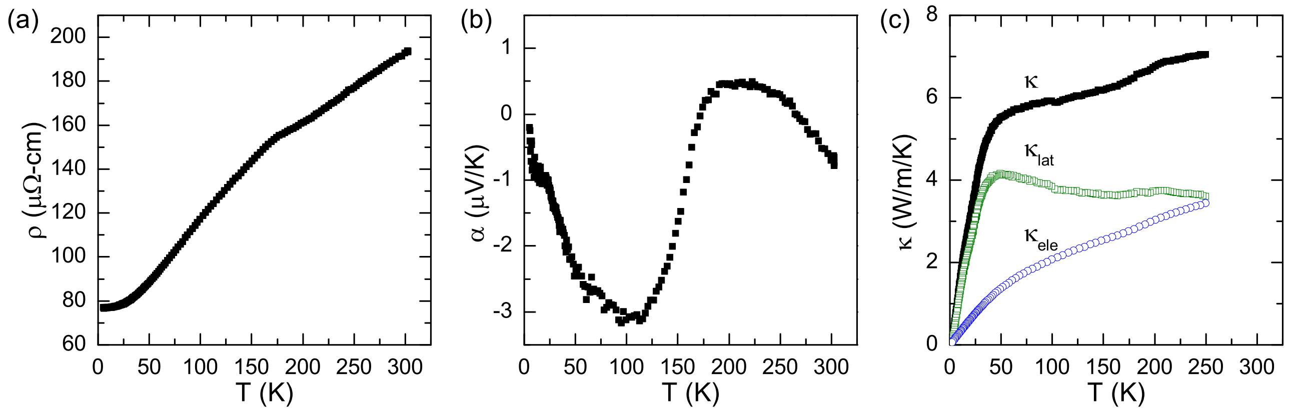

Figure S3. (a) Electrical resistivity, (b) Seebeck coefficient and (c) thermal conductivity of polycrystalline (hot-pressed) $CuFe_2Ge_2$. In (c), the electronic contribution has been subtracted from the total thermal conductivity κ to yield an estimate for the lattice contribution κ_{lat}.

applied force of 500 kg; the temperature was measured using a thermocouple inserted into the body of the die. This yielded a pellet of approximately 87% of the theoretical density, which is not as dense as desired for some transport measurements but is sufficient to provide insight into the connection between magnetism and transport. In particular, the density should have little influence on the behavior of the Seebeck coefficient, which is not influenced by the magnitude of the electron relaxation time but rather by its energy dependence. Thermal and electrical transport measurements were performed using the Thermal Transport Option of the Quantum Design PPMS. Gold-coated copper leads were attached using silver epoxy.

Electrical and thermal transport measurements were performed to examine how the itinerant magnetism influenced the other physical properties, and the results are shown in Fig. S3. The electrical resistivity displays a small change in slope near $T_N \approx$175 K, but does not show any significant response at the onset of incommensurate spin density wave near 100-125 K or at the onset of the enhanced magnetization near 230 K. The change in slope at T_N is commonly observed across magnetic transitions, and likely indicates a reduced scattering of charge carriers below T_N due to the loss of scattering from spin fluctuations.

The Seebeck coefficient (α) responds to all three magnetic ordering temperatures, as shown in Fig. S3b. A slope change is observed near $T_C \approx$ 230 K, followed by a strong change at T_N that leads to a sign change, and finally an extremum is observed at $\approx$100 K. The Seebeck coefficient is influenced by the shape of the Fermi surface and the scattering mechanisms of the free carriers. As such, it can be a sensitive probe of the electrical transport. In the simplest model, a positive Seebeck coefficient indicates that holes dominate conduction while a negative Seebeck coefficient indicates electrons dominate conduction. Thus, the change in sign of α is likely associated with a redistribution of the electronic states upon transitions between various magnetic states, and also indicates that multiple bands contribute to the Fermi surface.

The thermal conductivity is shown in Fig. S3c. Also shown are estimates for the lattice contribution $\kappa_{lat} = \kappa - \kappa_{ele}$ and the electronic contribution obtained using the Wiedemann-Franz law, κ_{ele}=LT/ρ; the degenerate (metallic) limit of the Lorenz number L=2.44 $\times$ 10^{-8} W/Ω/K^2 was utilized. The data in Fig. S3c are limited to 250 K, because radiation effects can cause erroneous data at higher temperatures.

Above $\approx$ 50 K, the thermal conductivity increases with increasing temperature due to the increase in κ_{ele}. In this same temperature range, the lattice contribution only decreases slightly. This behavior deviates from the $1/T$ decay expected for crystalline lattice where phonon-phonon interactions dominate at high T. As such, this suggests that some other scattering mechanisms are suppressing κ. Given that κ_{lat} appears to change slightly near T_N, it may be reasonable to suggest that the phonons are scattered by magnetic excitation and/or fluctuations. Indeed, an interaction between thermal transport and magnetism is one additional manifestation of magnetoelastic coupling, consistent with the other observations of lattice-magnetism interactions. The relatively high porosity of the polycrystalline compact may also produce excess scattering that reduces κ_{lat} at all temperatures, though this would not lead to the small changes in κ near 170-200 K.

pXRD and Magnetism of $CuFeCoGe_2$

Cobalt doping was found to suppress the magnetism in $CuFe_2Ge_2$, allowing a comparison of the lattice properties in the magnetic $CuFe_2Ge_2$ to those in a non-magnetically-ordered material with similar chemical composition and structure. A sample of nominal composition $CuFeCoGe_2$ was arc-melted at least five times and the resulting ingot was annealed at 600°C for 7 d; a

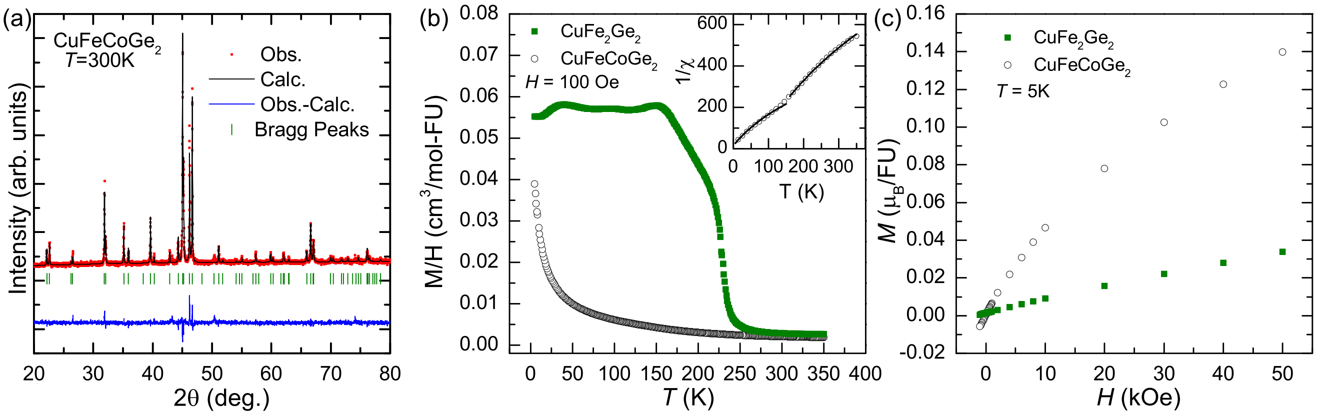

Figure S4. (a) Powder x-ray diffraction data for $CuFeCoGe_2$. (b) Temperature-dependence of the magnetization of $CuFeCoGe_2$ in comparison to $CuFe_2Ge_2$, with inset showing $1/\chi$ data for $CuFeCoGe_2$. (c) Isothermal magnetization of $CuFeCoGe_2$ and $CuFe_2Ge_2$ at 5 K.

piece of $CuFe_2Ge_2$ was made in the same way for a direct comparison. The original structure paper for $CuFe_2Ge_2$ reported a cobalt-based phase of the same structure type, though partial mixing of the Cu and Co sites were reported.[1] Powder x-ray diffraction data for $CuFeCoGe_2$ at 300 K are shown in Fig. S4a, along with a Rietveld refinement in the expected structure; these data show phase purity equivalent to that in the undoped $CuFe_2Ge_2$ samples.

Magnetization data for the $CuFeCoGe_2$ sample is compared to that of $CuFe_2Ge_2$ in Fig. S4b,c. The ferromagnetic-like onset of the magnetization near 228 K and the antiferromagnetic order are all suppressed by this nominal cobalt doping of 50%. When examining the behavior of $1/\chi$, where the susceptibility $\chi = M/H$, there is a slight kink in the data for $CuFeCoGe_2$ near 157(2) K. This subtle feature can also be observed in the derivative $d\chi/dT$ (not shown). The exact nature of this anomaly is not known. The isothermal magnetization is shown in Fig. S4c, which shows the cobalt-containing sample to be more easily polarized compared to the antiferromagnetically-ordered $CuFe_2Ge_2$. This may suggest that finer control of the cobalt doping could lead to a ferromagnetic ground state, though a detailed study into the physical properties of cobalt-doped samples should be performed before any conclusions are drawn. In any case, $CuFeCoGe_2$ appears to be a suitable material to utilize as a non-magnetically-ordered analogue when considering the evolution of the lattice parameters as a function of T in $CuFe_2Ge_2$.

First principles calculations

First principles calculations were utilized to explore the magnetoelastic coupling and to aid in the refinement of neutron diffraction data. In general, our calculation results are in agreement with those presented by Shanavas and Singh.[2] To provide a more direct comparison with the Fe-based superconductor systems, which also display significant magnetoelastic coupling, the magnetic moments were calculated as a function of the Fe(2) z coordinate. An increase in this z represents a reduction in the Fe(1)-Fe(2) bond distance, and a further spacing of the Fe(2)-Fe(2) distance that separates the sawtooth chains of Fe. The calculation results are shown in Fig. S5, and opposite trends for the two Fe sites are observed. In general, though, the change is not as significant as that observed in the Fe-based superconducting materials. These calculations were done within the AF0 ground state, as described in the Methods Section of the main text.

The coupling between the imposed magnetic structure and the crystal structure was also investigated with first principles calculations. The unconstrained atomic coordinates (z of Ge(1), Ge(2), and Fe(2)), were relaxed after imposing a given magnetic structure. The results are summarized in Table S1. This calculation revealed that the ferromagnetic configuration, which we obtain to have the largest moments, produces the greatest response in the lattice. This calculation also reveals the proximity of the various magnetic ground states.

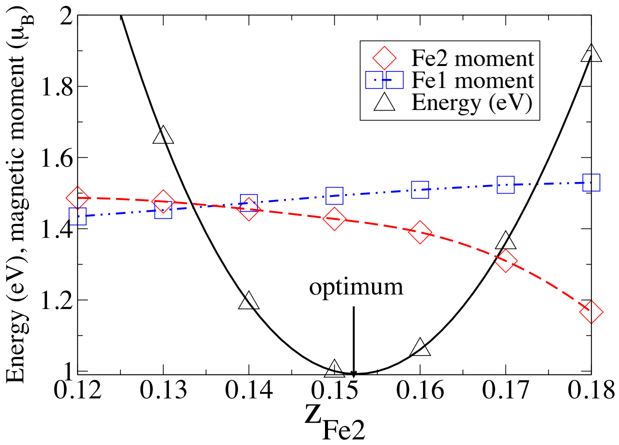

Figure S5. Calculation probing the influence of the Fe(2) z-coordinate on the Fe(1) and Fe(2) moments, as well as the total energy within the calculated AF0 ground state.

Table S1. Calculated results for the non-magnetically-ordered state and several magnetic states.

State	z_{Fe_2}	Δ E (meV/Fe)	Staggered moment Fe(1), Fe(2)
Non-magnetic	00.1541	-	-
Ferromagnetic	00.1636	-58	+10.55, +10.50
AF1	00.1524	-84	$\pm$ 1.32, $\pm$ 1.32
AF0	00.1521	-99	$\pm$ 1.49, $\pm$ 1.41

X-ray and Neutron Diffraction Data and Analysis

To complement the temperature-dependent neutron diffraction results, powder x-ray diffraction data at T=200, 160, and 60 K are presented in Figure S6. These results demonstrate that the nuclear structure does not fundamentally change across the magnetic transitions. As noted in the main text, however, the c lattice parameter clearly responds to the magnetic transitions.

Figure S7 shows the refined values of the unconstrained atomic coordinates for $CuFe_2Ge_2$ obtained from powder x-ray diffraction data. The corresponding lattice parameters are shown in Figure 4 of the main text. Data for two $CuFe_2Ge_2$ samples are shown, as is also shown in Figure 4 of the main text, along with data obtained for the sample of nominal composition $CuFeCoGe_2$. As observed in Fig. S7, the atomic coordinates are relatively independent of temperature and fairly similar for the different samples. Higher quality diffraction data, ideally from single crystals, would be required to better isolate any small shifts in atomic positions that occur in relation to the magnetic transitions.

Complete neutron diffraction datasets were collected at several temperatures, and data are shown for three pertinent temperatures in Figure S8. Data collected at $T = 200K$, which is below the ferromagnetic-like onset at 228 K, were well-described using the non-magnetic structure utilized for refinement of x-ray diffraction data. The data at $T = 135$ K were refined using the magnetic propagation vector $\mathbf{k}_C$=(0,$\frac{1}{2}$,0), and the inset of Figure S8b highlights the contribution of magnetic scattering. The magnetic intensity of this commensurate *AFM-C* phase was greater at 135 K than any other temperature examined in detail. The lower T, incommensurate structure *AFM-IC* with propagation vector $\mathbf{k}_{IC}$=(0,$\frac{1}{2}$,x) was utilized to model the data collected at 4 K. The non-indexed peak near 22 degrees 2θ in Figure S8 likely comes from alloys in the Al can, though it could also be due to a minor impurity within the sample.

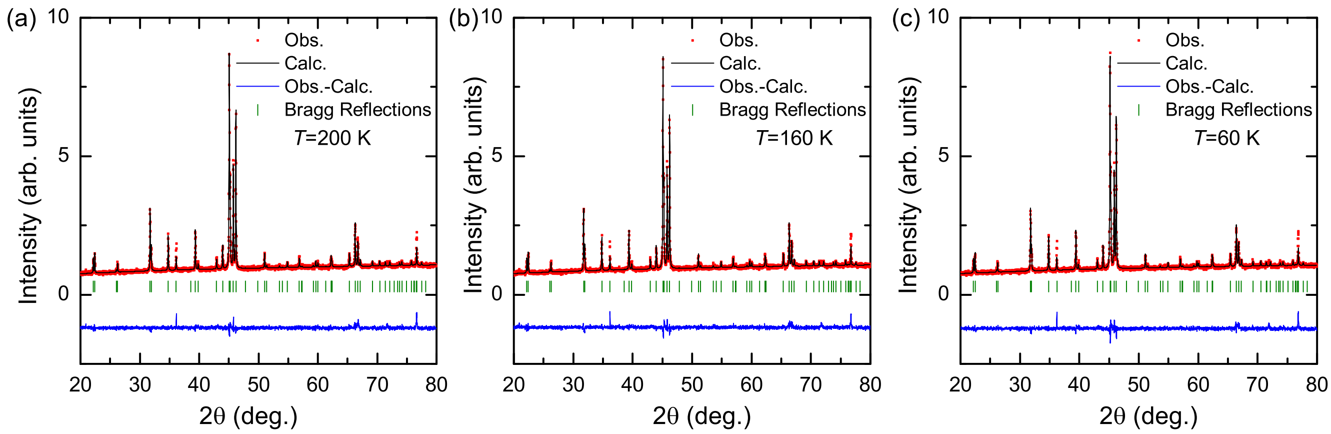

Figure S6. X-ray diffraction data and Rietveld refinements at (a) 200 K, (b) 160 K, and (c) 60 K.

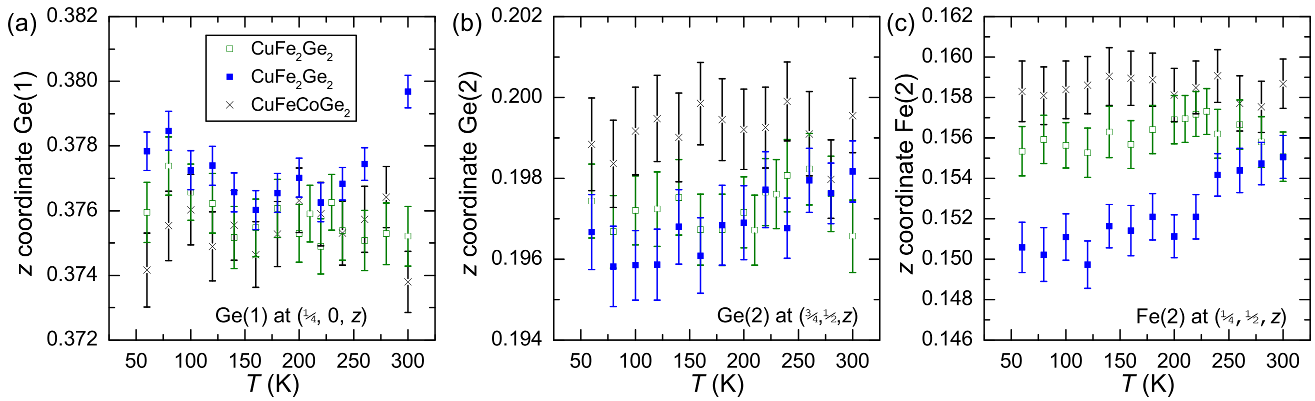

Figure S7. Atomic coordinates from Rietveld refinements of x-ray diffraction data for $CuFe_2Ge_2$ and $CuFeCoGe_2$. As in the main text, data for two different $CuFe_2Ge_2$ samples are shown (the open symbols correspond to the sample used for neutron diffraction).

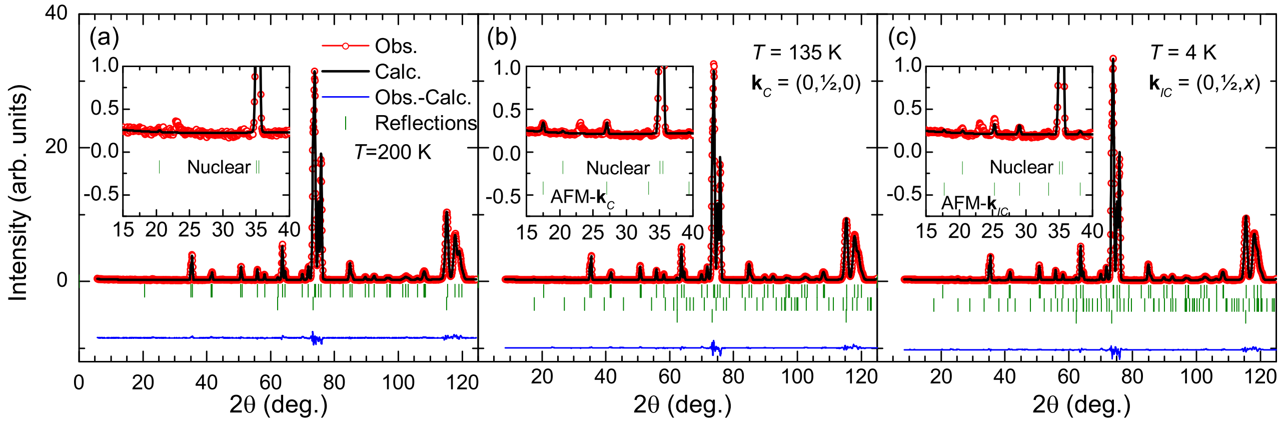

Figure S8. Neutron diffraction data and Rietveld refinements at (a) 200 K, (b) 135 K, and (c) 4 K. The inset highlights the region where magnetic scattering is indexed, with propagation vectors $\mathbf{k}_C$ and $\mathbf{k}_{IC}$ provided in the legend.

Representational Analysis T=135 K

Table S2. Basis vectors for the space group *Pmma* with $\mathbf{k}_C = (0,\ 0.5,\ 0)$.The decomposition of the magnetic representation for the *Fe* site $(0,\ 0.5,\ 0.5)$ is $\Gamma_{Mag} = 0\Gamma_1^1 + 1\Gamma_2^1 + 0\Gamma_3^1 + 2\Gamma_4^1 + 0\Gamma_5^1 + 1\Gamma_6^1 + 0\Gamma_7^1 + 2\Gamma_8^1$. The atoms of the nonprimitive basis are defined according to 1: $(0,\ 0.5,\ 0.5)$, 2: $(0.5,\ 0.5,\ 0.5)$.

IR	BV	Atom	BV components					
			$m_{\parallel a}$	$m_{\parallel b}$	$m_{\parallel c}$	$im_{\parallel a}$	$im_{\parallel b}$	$im_{\parallel c}$
Γ_2	ψ_1	1	0	4	0	0	0	0
		2	0	4	0	0	0	0
Γ_4	ψ_2	1	4	0	0	0	0	0
		2	4	0	0	0	0	0
	ψ_3	1	0	0	4	0	0	0
		2	0	0	-4	0	0	0
Γ_6	ψ_4	1	0	4	0	0	0	0
		2	0	-4	0	0	0	0
Γ_8	ψ_5	1	4	0	0	0	0	0
		2	-4	0	0	0	0	0
	ψ_6	1	0	0	4	0	0	0
		2	0	0	4	0	0	0

Table S3. Basis vectors for the space group *Pmma* with $\mathbf{k}_C = (0,\ 0.5,\ 0)$.The decomposition of the magnetic representation for the *Fe* site $(.25,\ 0.5,\ 0.15507)$ is $\Gamma_{Mag} = 1\Gamma_1^1 + 1\Gamma_2^1 + 1\Gamma_3^1 + 1\Gamma_4^1 + 1\Gamma_5^1 + 0\Gamma_6^1 + 0\Gamma_7^1 + 1\Gamma_8^1$. The atoms of the nonprimitive basis are defined according to 1: $(0.25,\ 0.5,\ 0.15507)$, 2: $(0.75,\ 0.5,\ 0.84493)$.

IR	BV	Atom	BV components					
			$m_{\parallel a}$	$m_{\parallel b}$	$m_{\parallel c}$	$im_{\parallel a}$	$im_{\parallel b}$	$im_{\parallel c}$
Γ_1	ψ_1	1	4	0	0	0	0	0
		2	-4	0	0	0	0	0
Γ_2	ψ_2	1	0	4	0	0	0	0
		2	0	4	0	0	0	0
Γ_3	ψ_3	1	0	4	0	0	0	0
		2	0	-4	0	0	0	0
Γ_4	ψ_4	1	4	0	0	0	0	0
		2	4	0	0	0	0	0
Γ_5	ψ_5	1	0	0	4	0	0	0
		2	0	0	-4	0	0	0
Γ_8	ψ_6	1	0	0	4	0	0	0
		2	0	0	4	0	0	0

Representational Analysis T=4 K

Table S4. Basis vectors for the space group *Pmma* with $\mathbf{k}_{IC} = (0,\ 0.5,\ 0.125)$.The decomposition of the magnetic representation for the *Fe* site $(0,\ 0.5,\ 0.5)$ is $\Gamma_{Mag} = 2\Gamma_1^1 + 1\Gamma_2^1 + 1\Gamma_3^1 + 2\Gamma_4^1$. The atoms of the nonprimitive basis are defined according to 1: $(0,\ 0.5,\ 0.5)$, 2: $(0.5,\ 0.5,\ 0.5)$.

IR	BV	Atom	BV components					
			$m_{\parallel a}$	$m_{\parallel b}$	$m_{\parallel c}$	$im_{\parallel a}$	$im_{\parallel b}$	$im_{\parallel c}$
Γ_1	ψ_1	1	2	0	0	0	0	0
		2	2	0	0	0	0	0
	ψ_2	1	0	0	2	0	0	0
		2	0	0	-2	0	0	0
Γ_2	ψ_3	1	0	2	0	0	0	0
		2	0	2	0	0	0	0
Γ_3	ψ_4	1	0	2	0	0	0	0
		2	0	-2	0	0	0	0
Γ_4	ψ_5	1	2	0	0	0	0	0
		2	-2	0	0	0	0	0
	ψ_6	1	0	0	2	0	0	0
		2	0	0	2	0	0	0

Table S5. Basis vectors for the space group *Pmma* with $\mathbf{k}_{IC} = (0,\ 0.5,\ 0.125)$.The decomposition of the magnetic representation for the *Fe* site $(.25,\ 0.5,\ 0.15533)$ is $\Gamma_{Mag} = 1\Gamma_1^1 + 1\Gamma_2^1 + 0\Gamma_3^1 + 1\Gamma_4^1$. The atom of the primitive basis is defined according to 1: $(.25,\ 0.5,\ 0.15533)$.

IR	BV	Atom	BV components					
			$m_{\parallel a}$	$m_{\parallel b}$	$m_{\parallel c}$	$im_{\parallel a}$	$im_{\parallel b}$	$im_{\parallel c}$
Γ_1	ψ_1	1	4	0	0	0	0	0
Γ_2	ψ_2	1	0	4	0	0	0	0
Γ_4	ψ_3	1	0	0	4	0	0	0

Table S6. Basis vectors for the space group *Pmma* with $\mathbf{k}_{IC} = (0,\ 0.5,\ 0.125)$.The decomposition of the magnetic representation for the Fe_2 site $(0.75,\ 0.5,\ 0.84467)$ is $\Gamma_{Mag} = 1\Gamma_1^1 + 1\Gamma_2^1 + 0\Gamma_3^1 + 1\Gamma_4^1$. The atom of the primitive basis is defined according to 1: $(0.75,\ 0.5,\ 0.84467)$.

IR	BV	Atom	BV components					
			$m_{\parallel a}$	$m_{\parallel b}$	$m_{\parallel c}$	$im_{\parallel a}$	$im_{\parallel b}$	$im_{\parallel c}$
Γ_1	ψ_1	1	4	0	0	0	0	0
Γ_2	ψ_2	1	0	4	0	0	0	0
Γ_4	ψ_3	1	0	0	4	0	0	0

References

1. Zavalij, I. Crystal structures of $CuFe_2Ge_2$ and $Cu_{1+/-x}Co_{2-/+x}Ge_2$. *Kristallografia* **32**, 66 (1987).

2. Shanavas, K. V. & Singh, D. J. Itinerant magnetism in metallic $CuFe_2Ge_2$. *PLoS ONE* **10**, 1–9 (2015). URL http://dx.doi.org/10.1371%2Fjournal.pone.0121186.
